# Effects of Oil-Contaminated Sediments on Submerged Vegetation: An Experimental Assessment of *Ruppia maritima*

**DOI:** 10.1371/journal.pone.0138797

**Published:** 2015-10-02

**Authors:** Charles W. Martin, Lauris O. Hollis, R. Eugene Turner

**Affiliations:** Department of Oceanography & Coastal Sciences, School of the Coast and Environment, Louisiana State University, Baton Rouge, Louisiana, United States of America; INRA, FRANCE

## Abstract

Oil spills threaten the productivity of ecosystems through the degradation of coastal flora and the ecosystem services these plants provide. While lab and field investigations have quantified the response of numerous species of emergent vegetation to oil, the effects on submerged vegetation remain uncertain. Here, we discuss the implications of oil exposure for *Ruppia maritima*, one of the most common species of submerged vegetation found in the region affected by the recent Deepwater Horizon oil spill. We grew *R*. *maritima* in a range of manipulated sediment oil concentrations: 0, 0.26, 0.53, and 1.05 mL oil /L tank volume, and tracked changes in growth (wet weight and shoot density/length), reproductive activity (inflorescence and seed production), root characteristics (mass, length, diameter, and area), and uprooting force of plants. While no statistical differences were detected in growth, plants exhibited significant changes to reproductive output, root morphology, and uprooting force. We found significant reductions in inflorescences and fruiting bodies at higher oil concentrations. In addition, the roots growing in the high oil were shorter and wider. Plants in medium and high oil required less force to uproot. A second experiment was performed to separate the effects of root morphology and oiled sediment properties and indicated that there were also changes to sediment cohesion that contributed to a reduction in uprooting forces in medium and high oil. Given the importance of sexual reproduction for these plants, oil contamination may have substantial population-level effects. Moreover, areas containing buried oil may be more susceptible to high energy storm events due to the reduction in uprooting force of foundation species such as *R*. *maritima*.

## Introduction

Coastal seagrasses and submerged aquatic vegetation are historically abundant throughout coastal regions worldwide [1–3]. Despite their widespread distribution, numerous threats such as disease, physical disturbance (such as boat propeller scars and hurricanes), eutrophication, sediment input, overharvesting of top predators leading to an increase in herbivores, and global climate change have all been posited to have reduced the areal coverage of submerged vegetation to historically low levels [4–5]. In Louisiana (USA), for example, there was a 50–75% decline in macrophyte coverage between 1954 and 1992 [6–8].

The estuaries of the northern Gulf of Mexico (GoM) are relatively productive areas [9–10] because of a diverse mix of submerged and emergent vegetation. These structured habitats provide refuge for the young of many commercially and recreationally important nekton species [11–13], contribute to the forage base of consumers [14–15], buffer coastlines from high energy events [16–18], and improve water quality and clarity to potentially mitigate some of the negative effects of eutrophication [19–21].

The 2010 Deepwater Horizon (DWH) oil spill in the GoM has the potential to greatly exceed all other regional threats to coastal vegetation. Approximately 4.9 million barrels of oil [22] were released over the course of 87 days, prompting a number of protective measures to keep oil out of vital wetland areas including: application of chemical dispersant both at depth and aerially to increase the surface area of oil and enhance microbial degradation, opening river diversions to increase freshwater discharge and possibly keeping oil offshore, burning oil at the water’s surface, and mobilizing cleanup crews and local fishermen to place oil protection booms and remove oil from coastlines [23–24]. Despite these efforts, there was oiling of 1,773 km of shoreline, over 60% of which occurred along the Louisiana coast [24].

Experimental studies conducted to date have focused on the oil’s impacts to emergent vegetation such as *Spartina alterniflora*, *S*. *patens*, *Juncus roemerianus*, and *Phragmites australis*. The results of oil exposure experiments have been highly variable and species-specific [25], with some species, such as *S*. *patens* [26–27] more susceptible than others. Some species of emergent vegetation, such as *P*. *australis* [28], *Sagittaria lancifolia* [26,29], and in a few cases *S*. *alterniflora* [30–34], appears to be resilient to the toxic effects of oil. Physical smothering of plant tissue reducing photosynthesis, application of oil to soils, and repeated, heavy exposure seems to have a large impact on plant productivity [28,35]. The effects of oil on submerged vegetation (such as seagrasses and other subtidal, coastal species), however, remain untested, perhaps because many researchers assume that the oil floated over grassbeds. Moreover, many submerged grasses are ephemeral and field studies to understand disappearance of these grasses are difficult to design without controlled, manipulative experimentation.

Findings from previous spills such Ixtoc-I [36], Exxon Valdez [37–38], and the *Florida* barge in Buzzards Bay, MA [39], as well as this spill [40–41], indicate that oil can persist in the environment buried in the sediment where minimal weathering occurs [42] and continue to affect coastal flora and fauna long after the spill [24,39,43]. Additional evidence suggesting that Macondo oil may be buried in nearshore environments is that marshes in Barataria Bay, Louisiana (USA) were re-oiled with remobilized Macondo oil after Hurricane Isaac in September 2012 [24,44]. We hypothesize, therefore, that oiled sediments will influence submerged vegetation and influence assessments and restoration efforts of benthic areas [28]. Here, we present the results of a manipulative experiment to determine the effects of varying concentrations of oil within sediments on one cosmopolitan species of submerged vegetation found throughout the northern GoM.

## Methods

We performed a series of experiments to determine the impacts of oil on a common submerged macrophyte species, widgeon grass (*Ruppia maritima* L. sensu lato, hereafter referred to as *Ruppia*). In Experiment 1, we exposed plants to various experimental doses of oil under greenhouse conditions and measured the consequences to plant growth, reproduction, morphology, and uprooting forces. In Experiment 2, uprooting force was tested independent of plants. The effect of oil on sediment cohesion was tested by burying inert substances (plastic beads) in the various oil concentrations and measuring the force needed to remove beads from the sediment.

### Study Species

*Ruppia* is among the most widespread species of submerged vegetation found in GoM estuaries [45–47], especially in Louisiana [7,48–49]. *Ruppia* is thought to be very tolerant of fluctuating environmental conditions and is found in areas highly variable in salinity. As a result, the distribution of *Ruppia* is thought to be governed by competition with other submerged macrophytes [45,50]. *Ruppia* is used as a food source by a variety of herbivores from benthic invertebrates [51] to fishes [52] and waterbirds [53] and serves as habitat for numerous nekton species [49].

### Experiment 1: Oil Effects on *Ruppia maritima*

#### Experimental Design

We used *Ruppia* and sediments collected from Lake Pontchartrain near Lacombe, Louisiana (30.258, -89.948). No permits were required for the described study, which complied with all relevant regulations. In addition, no private land was accessed and this study involved no threatened or endangered species. Whole plants were placed in a cooler with an airstone and transported to the Louisiana State University Greenhouse Facility in Baton Rouge, LA, where the experiment took place. Approximately 1 L of sediment (3 cm) was placed in each 19 L tank, before adding a homogenous layer of oil (1 of 4 randomly selected levels: 0, 5, 10, or 20 mL; hereafter referred to as none, low, medium, and high, respectively), and then another 1 L (3 cm) of sediment was added. These concentrations were chosen because it represents a range of concentrations used in previous experimental studies [54–55]. The oil used in this experiment was obtained from the Marlin Platform of the Dorado field and contains an almost identical toxicity/chemistry as MC252 oil from the Deepwater Horizon drilling platform. We used unweathered oil in this experiment to represent a potential worst-case scenario. Each treatment was replicated 12 times. At the conclusion of the experiment, a sediment sample was taken from one tank in each treatment to verify oil concentrations using the GCMS methods described in Turner et al. [56–57].

Before planting, whole *Ruppia* plants were spun in a salad spinner for approximately 60 spins to remove epiphytes and potential herbivores. Any remaining epiphytes were removed by hand without damaging the plant [48]. Three individual plants were then planted in each tank to approximately 3 cm depth. Dechlorinated 10 psu seawater, mixed using Instant Ocean ® salt mix, filled tanks to their 19 L capacity. The surface water temperatures ranged between 23–31°C during the experiment. All tanks contained an airstone, 10% of the water was changed every other day, and water was added daily to account for evaporation.

The tanks were planted/harvested on 4 different dates and each treatment was represented equally on each date). Tanks were planted from July 24, 2014 through August 6, 2014 and the experiment was terminated after 31–33 days.

#### Growth and Reproductive Activity

Several indicators of growth were measured before and after the experiment. Before planting, we measured the wet weight of plants on an Ohaus ® Model TS400S balance (± 0.01 g). Shoot density was determined as the number of separate, vertical stems ascending from the rhizome. The length of each shoot was measured (± 1 mm), and reproductive activity determined by counting the number of inflorescences and fruiting bodies on each plant. At the conclusion of the experiment, all plants were again placed in the salad spinner and these same variables were quantified. The data were standardized to a per day basis in the analyses to account for the slight variation in trial length. Proportional change was calculated as the (X_final_-X_initial_)/X_initial_, where X represents one of the aforementioned response variables.

#### Root Morphology

We hypothesized that roots may show oil-induced effects, because roots were in closest proximity to the oil layer. Therefore, a more detailed analysis of roots was determined for one randomly selected plant in each tank at the conclusion of the experiment. Three randomly selected roots from each plant were removed using a scalpel for measurement. Root length was measured by securing the specimen to a board with rubber bands. The total length of an individual root was measured using a digital caliper (± 0.1 mm) and digital planimeter (Calculated Industries Scale Master® Classic v3.2 Model 6020). A digital micrometer (Starrett® IP67) secured to a Bunsen burner stand with clamps was used to measure root diameter at three points along the root to the nearest (± 0.01 mm). Mass was measured by weighing each root on a digital scale (Mettler Toledo AB104S; ± 0.1 mg). Root cross-sectional area was calculated by assuming that the roots were cylindrical and using the formula for the area of a circle [A = ∏(d/2)^2^]. Volume was calculated by multiplying the cross-sectional area by the length of the root.

#### Uprooting Force

We measured the force needed to uproot one randomly selected plant in each tank at the end of the experiment using a Lyman® digital pull gauge, a technique used in previous studies [58–60]. A clip, tied to a line to the gauge, was placed at the base of each plant and vertical pressure exerted until the plant was removed from the sediment. The maximum amount of force (± 0.02 N) was recorded.

### Experiment 2: Soil Cohesion

A second experiment was conducted to identify the oiling effects on sediment cohesion independent of plants. This experiment was conducted with identical treatments and conditions, with the exception that instead of plants, five 12 mm plastic beads, each attached to a separate monofilament line, were placed in the oiled layer in each tank. At one week intervals, the force needed to remove one bead from the sediment in each tank was measured using the Lyman® digital pull gauge as before.

### Statistical Analyses

Assumptions (normality and homogeneity of variance) were tested before analysis, and data were transformed if the assumptions were not satisfied. The daily proportional change in wet weight, stem number, stem length, inflorescences, and fruiting bodies were analyzed using a General Linear Model with oil treatment (none, low, medium, and high) as a fixed factor and planting date as a random factor [61]. Root measurements (mass, length, diameter, and area) and uprooting force, both attained only at the conclusion of the experiment, were also analyzed using this approach. The bead experiment was analyzed using a repeated measures analysis of variance. All values were considered significant at p≤ 0.05 and Tukey’s post hoc test conducted when significant differences were detected. Statistical analyses were performed in MINITAB v13.

## Results

### Experiment 1: Oil Effects on *Ruppia maritima*

Chemical analyses of a subset of tanks verified the presence of numerous compounds associated with oil contamination (S1 and S2 Figs). These concentrations are within the range of those found in field conditions after the DWH oil spill [56–57].

#### Growth and Reproductive Activity

**Fig 1:**
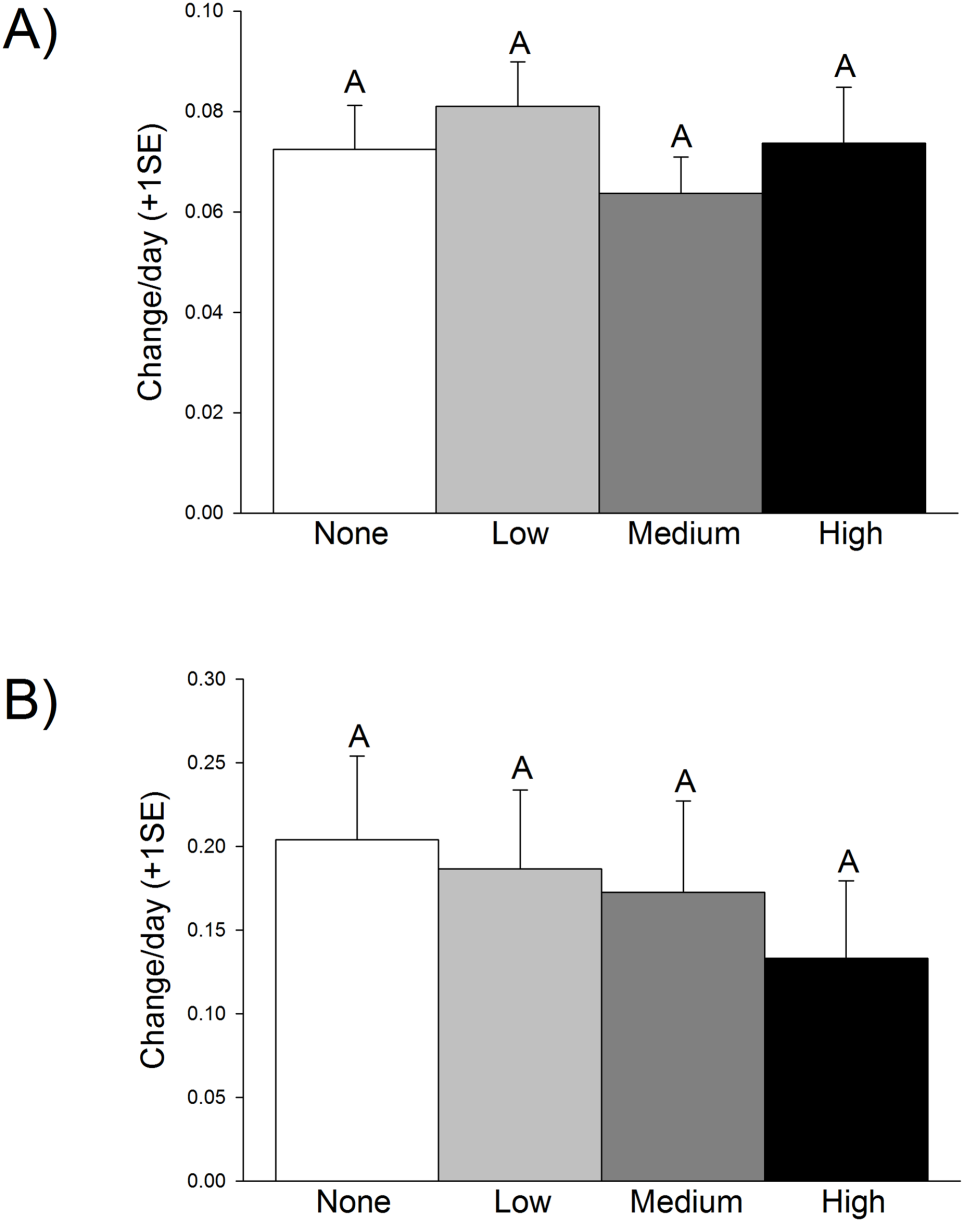
Daily proportional change for (A) wet weight, which increased from an initial average biomass of 0.31 g to 1.09 g, and (B) stem density, which increased from 3.7 to over 5 shoots per tank, for plants grown in the four different oil treatments: none (white), low (gray), medium (dark gray), and high (black). Letters indicate statistically-significant results. There were no statistically-significant differences among treatments (N = 12 per treatment).

*Ruppia* grew in all treatments, with little differences found in growth (Table 1). Plants grew from an initial biomass of approximately 0.31 g to an average of 1.09 g over the course of the experiment. This growth rate is a daily proportional change of approximately 0.07, with no significant difference among oil treatments (Fig 1A). Likewise, stem density per plant increased from around 3.7 shoots initially to over 5 at the end of the experiment, which is a daily proportional change of approximately 0.17, with a tendency of decreasing density with increasing oil, although this trend was not significant (Fig 1B). Stem length was highly variable and not significant across treatments, with average tank stem length increasing from 15.0 cm to 21.7 cm over the experiment duration. This translates to a 69% increase in shoot length with an average growth rate of 0.2 cm per day.

**Table 1:** Results of a General Linear Model analysis of the various comparisons of growth, reproduction, and uprooting in the four different oil treatments. Statistically-significant values are in **bold**.

		Oil Treatment		
Response Variable	Transformation	F	df	p
Wet weight	Fourth Root	0.81	3	0.494
Shoot density	N/A	0.42	3	0.737
Stem length	N/A	0.88	3	0.461
Inflorescences	N/A	4.51	3	**0.008**
Fruiting bodies	N/A	3.93	3	**0.015**
Uprooting	N/A	15.6	3	**<0.001**

**Fig 2:**
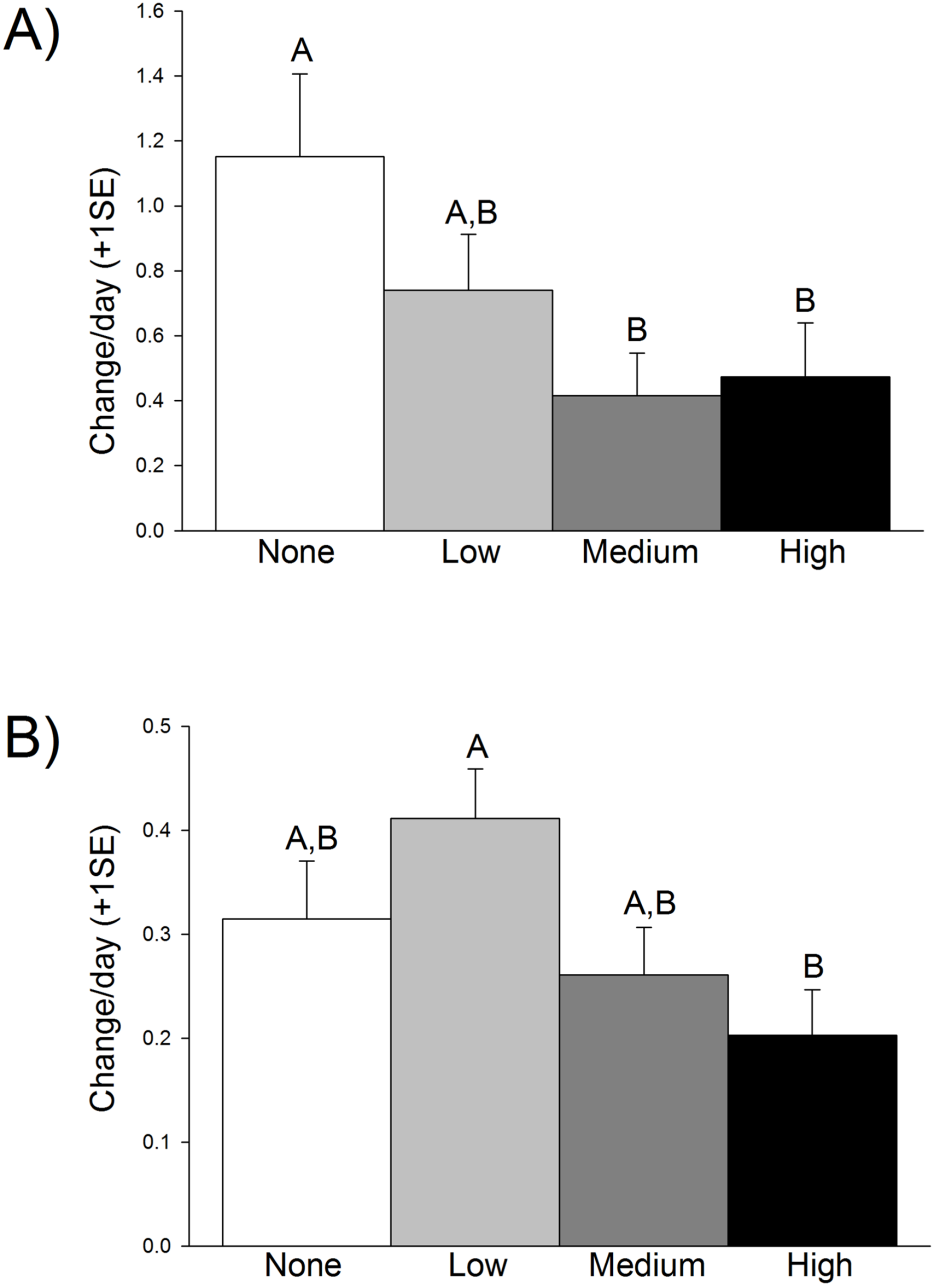
Reproductive output, expressed as daily proportional change. Fruiting bodies (A) increased from an average of 0 to 12.42 in no oil, 7.92 in low oil, 4.5 in medium oil, and 5.13 in high oil. Inflorescence production (B) increased from 1.2 flowers per tank to 3.6, 5.0, 3.2, and 2.6 inflorescences per plant in the none, low, medium, and high oil treatments, respectively, at the conclusion of the experiment. Letters indicate statistically-significant results (N = 12 per treatment).

Reproductive output, unlike growth, did differ significantly across oiling treatments (Table 1). The presence of fruiting bodies on plants significantly declined with oil concentration (Table 1, Fig 2A). Specifically, no oil significantly differed from medium (p = 0.019) and high oil (p = 0.034), while reproductive output in the low oil treatment was not different from any of the other treatment levels (p> 0.05). At the conclusion of the experiment, the number of fruits on plants changed from an approximately zero fruits found in all treatments to 12.42 in no oil, 7.92 in low oil, 4.5 in medium oil, and 5.13 in high oil. Expressed as the proportional change per day, this decrease was approximately 1.1, 0.75, 0.4, and 0.45 in none, low, medium, and high oil treatment, respectively. Likewise, inflorescences significantly differed across oiling treatments (Table 1, Fig 2B). With an average initial number of 1.15 flowers per tank, *post hoc* tests indicate that there were no differences between none, low, and medium oiling (p> 0.05), but flowering in the low oil treatment differed from the flowering in the high oil treatment (p = 0.006). The highest incidence of flowering was found in the low oil treatment, with a daily proportional change of around 0.4, decreasing to 0.31 in the no oil treatment and 0.25 in the medium treatment, and the lowest 0.20 in the high oil treatment. This translated to 3.6, 5.0, 3.2, and 2.6 inflorescences per plant in the none, low, medium, and high oil treatments, respectively, at the conclusion of the experiment.

#### Root Morphology

**Fig 3:**
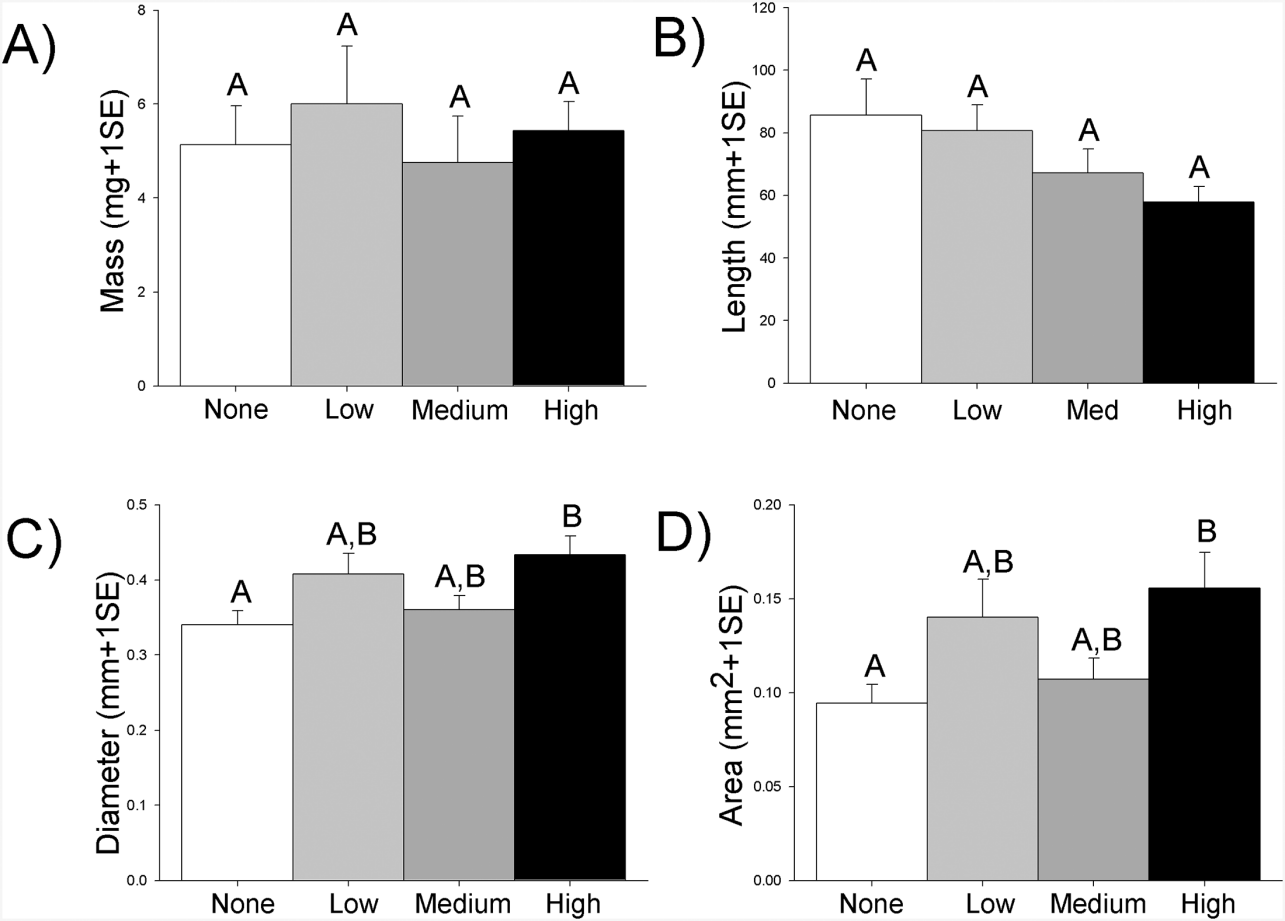
Changes in final root morphology across oil treatments [none (white), low (gray), medium (dark gray), and high (black)] for root mass (A), root length (B), root diameter (C), and root area (D). Letters indicate statistically-significant results (N = 12 per treatment).

The root morphology also changed significantly with oil exposure (Table 2, Fig 3). While root mass did not vary (Fig 3A), root length marginally decreased with increasing oil (Fig 3B). Roots were shortest in the high oil treatment (~0.58 mm), and increased in length in the medium (~0.65 mm), low (~0.80 mm), and no oil treatments (~0.83 mm). Conversely, roots were wider with increasing oil concentrations, with the average diameters ranging from 0.34 mm in the no oil treatment to 0.40 mm and 0.35 mm in low and medium treatments, and the largest diameters of 0.43 mm were in the high oil treatment (Fig 3C). This difference was driven by the diameters in the none and high oil treatments (p = 0.027; Table 3). Root area similarly increased across oiling, with areas of 0.09 mm^2^ in the none, 0.14 mm^2^ in low, 0.11 mm^2^ in medium, and 0.15 mm^2^ in high oil treatments (Fig 3D). Again, this increase was statistically driven by the significant difference between none and high oil (p = 0.017; Table 3).

**Table 2:** General Linear Model analysis of root morphology. Statistically-significant values are in **bold**.

		Oil Treatment		
Response Variable	Transformation	F	df	p
Mass	Fourth Root	0.5	3	0.686
Length	Fourth Root	2.66	3	0.061
Diameter	N/A	3.59	3	**0.021**
Area	Log	3.95	3	**0.015**

**Table 3:** Summary of significant changes in the treatment plots compared to control plots for different parameters. Only statistically-significant results are shown. n.s. = not significant.

	Oil Treatment		
Parameter	Low	Medium	High
Wet weight	n.s.	n.s.	n.s.
Stem density	n.s.	n.s.	n.s.
Stem length	n.s.	n.s.	n.s.
Seed production	n.s.	**0.019**	**0.034**
Inflorescence production	n.s.	n.s.	n.s.
Plant uprooting force	n.s.	**0.030**	**0.001**
Inert bead uprooting force	n.s.	**<0.001**	**<0.001**
Root mass	n.s.	n.s.	n.s.
Root length	n.s.	n.s.	n.s.
Root diameter	n.s.	n.s.	**0.027**
Root area	n.s.	n.s.	**0.017**

#### Uprooting Force

**Fig 4:**
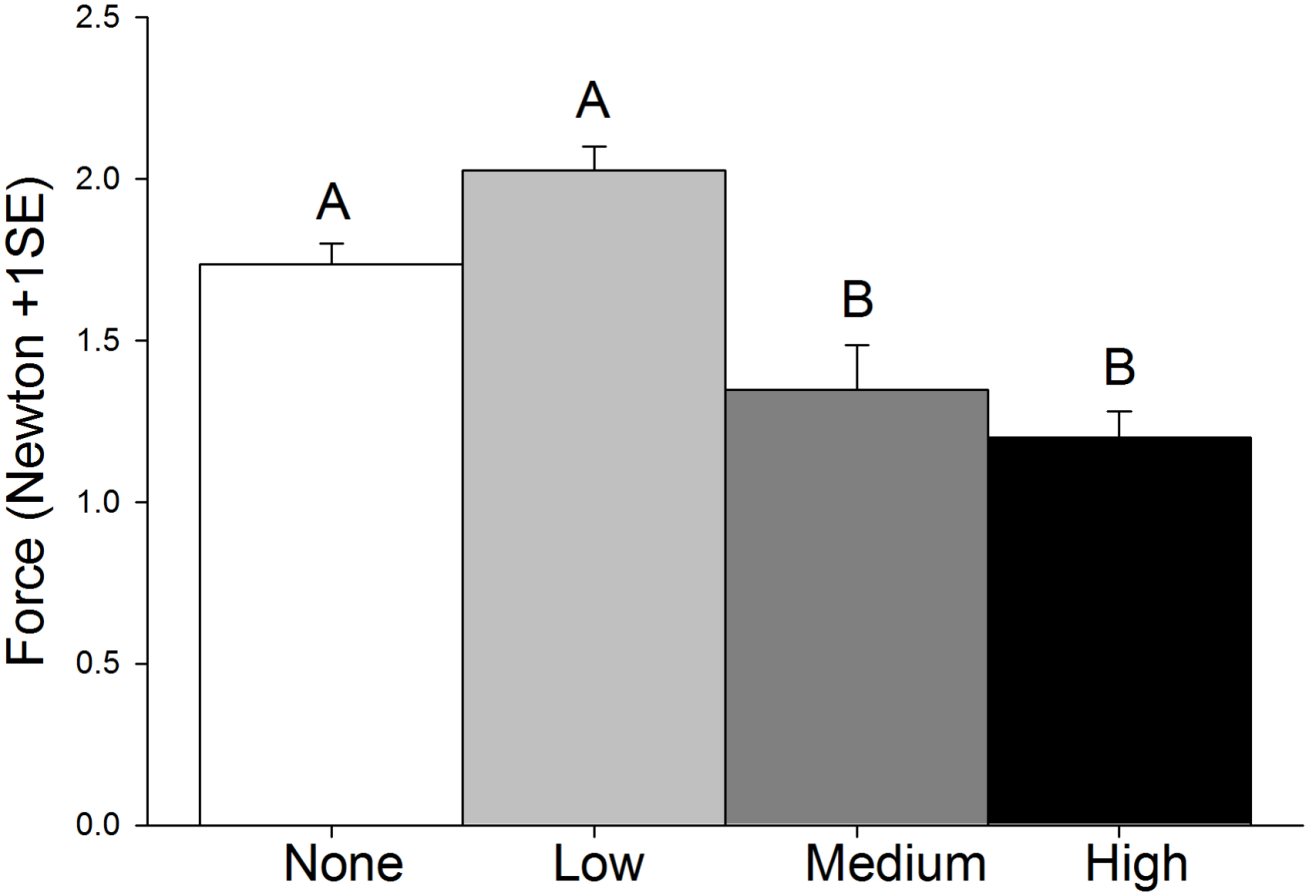
Difference in the uprooting force required to remove plants grown in four different oil treatments: none (white), low (gray), medium (dark gray), and high (black). Letters indicate statistically-significant results (N = 12 per treatment).

The force needed to remove plants from sediment varied significantly across oil treatments (Table 1, Fig 4). *Post hoc* comparisons confirmed that medium and high treatments were not significantly different from each other (p = 0.688), but both were different from low (p< 0.001 in both cases) and no (medium oil vs no oil: p = 0.030, high oil vs no oil: p = 0.001) oil treatments. Significantly less force was needed to remove plants in medium (~1.4 N) and high oil treatments (~1.2 N) than plants in none (~1.7 N) and low oil treatments (~2.0 N).

### Experiment 2: Soil Cohesion

**Fig 5:**
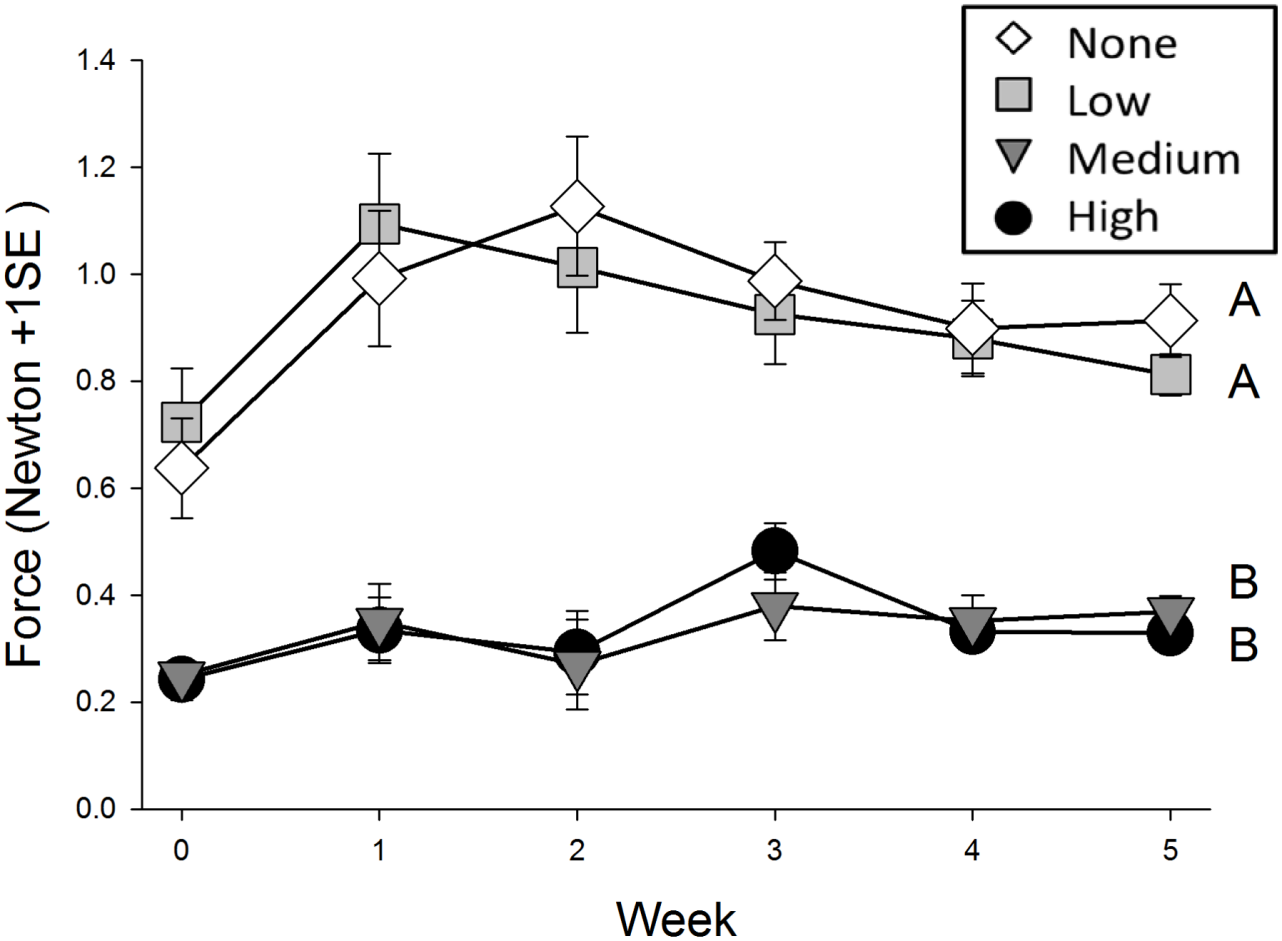
Changes in in the uprooting force required to remove inert plastic beads over time buried in the four different oil treatments: none (white diamond), low (gray square), medium (dark gray triangle), and high (black circle). Letters indicate statistically-significant results (N = 12 per treatment at each time interval).

An additional observation indicated that soil strength was significantly altered by oil (F_3,279_ = 106.51; p≤ 0.001) (Fig 5), with less force required to uproot beads in medium (~0.33 N) and high oil treatments (~0.33 N) than in the none (~0.93 N) and low oil treatment (~0.91 N). *Post hoc* tests indicated that soil cohesion in the medium and high oil treatments were statistically indistinguishable (p = 0.998), but both were different than low and medium oil treatments (p≤ 0.001). Low and medium oil levels were not significantly different from each other (p = 0.98). This trend was consistent over the 5 week course of the experiment.

## Discussion

The results of studies of the DWH incident’s impacts on GoM ecosystems have occasionally painted a picture of a resilient ecosystem, with a recovery of salt marshes [62], invertebrates [63], and fishes [64–65] in many oil-affected areas. However, other studies have noted the potential for long-term ecosystem alteration through continued exposure to hydrocarbon compounds [56–57] and a legacy of effects from increased erosion in the oiled areas [66]. Buried oil may represent one vector by which this spill may continue to alter the trajectory of ecosystem structure, function, and recovery [24,39,43]. Here, we found that buried oil affects the reproductive output, alters root morphology, and increases potential for erosion of one species of submerged vegetation, *R*. *maritima*. The implications of this study extend beyond the framework for assessing DWH oil spill impacts, because thousands of oil and natural gas structures currently exist in both offshore and nearshore regions throughout the northern GoM.

We found that the growth of *Ruppia* was resilient to the toxic effects of oil, with plants growing throughout the range of oil concentrations tested here. With many oil and natural gas structures throughout the area, as well as natural seeps, it is possible that resilient plants have already been selected for, and plants are previously adapted to, these conditions. Culbertson et al. [39], however, found that after four decades of exposure, *S*. *alterniflora* in Buzzards Bay still exhibited long-term impacts in areas where buried oil persists, with lower above- and below-ground biomass in oiled areas indicating that adaptation to these conditions has not occurred to date.

The reduction in reproductive output under high oil conditions may have consequences for some populations of *Ruppia*. Dunton [45], for example, found that some populations are entirely dependent on the seed bank as a mechanism for new growth, while other populations may maintain vegetative mechanisms of dispersal. Similarly, Cho and Poirrier [7] took monthly core samples from four areas containing *Ruppia* in Louisiana and found spatial and temporal variability in the amount of root and rhizome biomass that persisted throughout the winter, suggesting some reliance on sexual reproduction may be present for some populations. The impact of oil on seed germination and the seed bank remains the focus of future study.

*Ruppia* flowers opportunistically under favorable conditions [45] and this may explain the increase in flowering in unoiled and low oiled treatments. Conversely, some plants are known to increase flowering as a response to stress as a means of enhancing potential resistance to stressors through genetic recombination [59,67–69]. The decrease in *Ruppia* flowering at high oil levels could, therefore, indicate that the plant is less stressed and perhaps even using oil as a nutritional reserve. The consequences of these trends in reproduction require further investigation.

In several instances, oil has been hypothesized to be a carbon source for organisms. For example, carbon isotopes have been used to demonstrate the incorporation of depleted signatures into plankton [70] and deep sea nekton [71], because oil contains a depleted signature of approximately -27 δ^13^C. Lin and Mendelssohn [26] found that oil stimulated growth of *S*. *lancifolia* at concentrations up to 24 L m^-2^. While we found no differences in growth for *Ruppia*, the decrease in root length and increase in area may indicate that plant roots are able to use oil as a source of nutrition, thus making it unnecessary to grow deeper. Alternatively, it is possible that the redox conditions created by the oil layer [72] may influence this lack of vertical growth, although several studies [73–75] indicate that oxygen transported to the rhizomes and roots creates an oxic microzone in sediments around roots in submerged grasses.

Reductions in uprooting force indicate a vulnerability to physical forces for not only *Ruppia*, but all plants living in oil contaminated areas. While some of this is due to the change in root morphology, the second experiment, designed to tease apart the effects of root morphology and sediment cohesion, indicate that significant changes to the soil occur at the medium and high levels of oil used here. Plants, even in the medium and high oil treatments, required more force to uproot than do the inert beads, however, indicating that both root morphology and sediment properties are important in this trend. To date, this is the first study (that we are aware of) to demonstrate these findings experimentally. However, a number of field studies have indicated that areas where oil came ashore experienced enhanced erosion [62,66], and this may be due not only to the toxicity to vegetation, but also to changes in sediment properties.

We acknowledge several deficiencies in the current study that should be improved with additional research. *Ruppia* was grown under optimal greenhouse conditions with no epiphytes or herbivores and it is possible that the addition of multiple stressors may have non-additive effects on plants [4]. Moreover, the amount of buried oil in oil-affected areas remains unknown and the subject of future work, but early estimates indicated that a large amount of oil (~2 million barrels) released at the wellhead is unaccounted for [76]. A portion (4–31%) of this has been found in sediments surrounding the accident site, and it has been hypothesized that the rest may be heterogeneously distributed in sediments throughout the region [77]. Additional testing of sediments in field locations should continue to search for this oil, perhaps under newly-accreted sediment. Field verification in reproductive trends and uprooting of plants in oiled areas, as well as how buried oil affects other less tolerant vegetation, could shed light on the generality of these results. Finally, we used unweathered oil in this experiment, although it is possible that the weathered oil that reached the coastline could have a different effect. It is important to note, however, that many of the chemical constituents of oil presumed to precipitate quickly, such as naphthalenes, have been found in marsh ecosystems [56–57]. It is our hope that these findings further our understanding of how this tragedy affected coastal fauna and flora and contribute positively to the continued conservation of coastal ecosystems in the northern GoM.

## Supporting Information

S1 Fig Alkane Hydrocarbon Concentrations. The concentration of alkane petroleum hydrocarbons (μg g^-1^) measured at the end of the experiment.
(TIF)

S2 Fig Aromatic Hydrocarbon Concentrations. The concentration of aromatics petroleum hydrocarbons (ng g^-1^) measured at the end of the experiment.
(TIF)

## References

[1] Iverson RL, Bittaker HF. Seagrass distribution and abundance in eastern Gulf of Mexico coastal waters. Estuar Coast Shelf Sci. 1986; 22(5):577–602.

[2] Peneva E, Griffith JA, Carter GA. Seagrass mapping in the northern Gulf of Mexico using airborne hyperspectral imagery: a comparison of classification methods. J Coastal Res. 2008; 850–856.

[3] Martin CW, Valentine JF. Impacts of a habitat-forming exotic species on estuarine structure and function: an experimental assessment of Eurasian milfoil. Estuaries Coast. 2011; 34(2): 364–372.

[4] Orth RJ, Carruthers TJ, Dennison WC, Duarte CM, Fourqurean JW, Heck KL, et al A global crisis for seagrass ecosystems. Bioscience. 2006; 56(12): 987–996.

[5] Waycott M, Duarte CM, Carruthers TJ, Orth RJ, Dennison WC, Olyarnik S, et al Accelerating loss of seagrasses across the globe threatens coastal ecosystems. Proc Natl Acad Sci U S A. 2009; 106(30): 12377–12381. 10.1073/pnas.0905620106 19587236 PMC2707273

[6] Turner RE, Darnell R, Bond J. Changes in the submerged macrophytes of Lake Pontchartrain (Louisiana): 1954–1973. Northeast Gulf Sci. 1980; 4: 44–49.

[7] Cho HJ, Poirrier MA. Seasonal growth and reproduction of *Ruppia maritima* L. sl in Lake Pontchartrain, Louisiana, USA. Aquat Bot. 2005; 81(1): 37–49.

[8] Poirrier MA, Handley LR. Statewide summary of Louisiana. Seagrass status and trends in the northern Gulf of Mexico, 1940–2002. 2007; 60–71.

[9] Chesney EJ, Baltz DM, Thomas RG. Louisiana estuarine and coastal fisheries and habitats: perspectives from a fish's eye view. Ecol Appl. 2000; 10(2): 350–366.

[10] Lellis-Dibble KA, McGlynn KE, Bigford TE. Estuarine fish and shellfish species in US commercial and recreational fisheries: economic value as an incentive to protect and restore estuarine habitat. National Oceanic and Atmospheric Administration, National Marine Fisheries Service, Office of Habitat Conservation, Habitat Protection Division. 2008.

[11] Beck MW, Heck KL, Able KW, Childers DL, Eggleston DB, Gillanders BM, et al The Identification, conservation, and management of estuarine and marine nurseries for fish and invertebrates: a better understanding of the habitats that serve as nurseries for marine species and the factors that create site-specific variability in nursery quality will improve conservation and management of these areas. Bioscience 2001; 51(8): 633–641.

[12] Heck KL, Hays G, Orth RJ. Critical evaluation of the nursery role hypothesis for seagrass meadows. Mar Ecol Prog Ser. 2003; 253: 123–136.

[13] Rozas LP, Martin CW, Valentine JF. Effects of reduced hydrological connectivity on the nursery use of shallow estuarine habitats within a river delta. Mar Ecol Prog Ser. 2013; 492: 9–20.

[14] Meziane T, Tsuchiya M. Fatty acids as tracers of organic matter in the sediment and food web of a mangrove/intertidal flat ecosystem, Okinawa, Japan. Mar Ecol Prog Ser. 2000; 200: 49–57.

[15] Chaplin GI, Valentine JF. Macroinvertebrate production in the submerged aquatic vegetation of the Mobile—Tensaw Delta: effects of an exotic species at the base of an estuarine food web. Estuaries Coast. 2009; 32(2): 319–332.

[16] Desbonnet A, Lee V, Pogue P, Reis D, Boyd J, Willis J, et al Development of coastal vegetated buffer programs. Coast Manage. 1995; 23(2): 91–109.

[17] Barbier EB, Koch EW, Silliman BR, Hacker SD, Wolanski E, Primavera J, et al Coastal ecosystem-based management with nonlinear ecological functions and values. Science. 2008; 319(5861): 321–323. 10.1126/science.1150349 18202288

[18] Das S, Vincent JR. Mangroves protected villages and reduced death toll during Indian super cyclone. Proc Natl Acad Sci U S A. 2009; 106(18): 7357–7360. 10.1073/pnas.0810440106 19380735 PMC2678660

[19] Childers DL, Day JW. Marsh-water column interactions in two Louisiana estuaries. II. nutrient dynamics. Estuaries. 1990; 13(4): 404–417.

[20] Tanner CC. Plants for constructed wetland treatment systems—a comparison of the growth and nutrient uptake of eight emergent species. Ecol Eng. 1996; 7(1): 59–83.

[21] Wu H, Zhang J, Li P, Zhang J, Xie H, Zhang B. Nutrient removal in constructed microcosm wetlands for treating polluted river water in northern China. Ecol Eng. 2011; 37(4): 560–568.

[22] Camilli R, Reddy CM, Yoerger DR, Van Mooy BA, Jakuba MV, Kinsey JC, et al Tracking hydrocarbon plume transport and biodegradation at Deepwater Horizon. Science. 2010; 330(6001): 201–204. 10.1126/science.1195223 20724584

[23] Peterson CH, Anderson SS, Cherr GN, Ambrose RF, Anghera S, Bay S, et al A tale of two spills: novel science and policy implications of an emerging new oil spill model. BioScience. 2012; 62(5): 461–469.

[24] Michel J, Owens EH, Zengel S, Graham A, Nixon Z, Allard T, et al Extent and degree of shoreline oiling: Deepwater Horizon oil spill, Gulf of Mexico, USA. PloS One. 2013; 8(6): e65087 10.1371/journal.pone.0065087 23776444 PMC3680451

[25] Pezeshki SR, Hester MW, Lin Q, Nyman JA. The effects of oil spill and clean-up on dominant US Gulf coast marsh macrophytes: a review. Environ Pollut. 2000; 108(2): 129–139. 15092943 10.1016/s0269-7491(99)00244-4

[26] Lin Q, Mendelssohn IA. A comparative investigation of the effects of south Louisiana crude oil on the vegetation of fresh, brackish and salt marshes. Mar Pollut Bull. 1996; 32(2): 202–209.

[27] Hester MW, Lin Q, Mendelssohn IA, DesRoches DJ. The potential for accelerated bioremediation and restoration of oil-impacted marshes through the selection of superior oil-tolerant vegetation (Final Report). US Department of Interior, Mineral Management Service, Gulf of Mexico OCS Regional Office, New Orleans, LA. 1998.

[28] Judy CR, Graham SA, Lin Q, Hou A, Mendelssohn IA. Impacts of Macondo oil from Deepwater Horizon spill on the growth response of the common reed *Phragmites australis*: a mesocosm study. Mar Pollut Bull. 2014; 79(1): 69–76. 24456856 10.1016/j.marpolbul.2013.12.046

[29] Dowty RA, Shaffer GP, Hester MW, Childers GW, Campo FM, Greene MC. Phytoremediation of small-scale oil spills in fresh marsh environments: a mesocosm simulation. Mar Environ Res. 2011; 52(3): 195–211. 10.1016/s0141-1136(00)00268-3 11570802

[30] DeLaune RD, Patrick WH, Buresh RJ. Effect of crude oil on a Louisiana *Spartina alterniflora* salt marsh. Environ Pollut. 1979; 20(1): 21–31.

[31] de la Cruz AA, Hackney CT, Rajanna B. Some effects of crude oil on a *Juncus* tidal marsh. J Elisha Mitchell Sci Soc Chapel Hill, N C. 1981; 97: 14–28.

[32] Smith CJ, Delaune RD, Patrick WH, Fleeger JW. Impact of dispersed and undispersed oil entering a Gulf Coast salt marsh. Environ Toxicol Chem. 1984; 3(4): 609–616.

[33] Pezeshki SR, De Laune RD. Effect of crude oil on gas exchange functions of *Juncus roemerianus* and *Spartina alterniflora* . Water, Air, and Soil Pollut. 1993; 68(3–4): 461–468.

[34] Lin Q, Mendelssohn IA. Impacts and recovery of the Deepwater Horizon oil spill on vegetation structure and function of coastal salt marshes in the northern Gulf of Mexico. Environ Sci Technol. 2012; 46(7): 3737–3743. 10.1021/es203552p 22369124

[35] Schrope M. The lost legacy of the last great oil spill. Nature News. 2010; 466(7304): 304–305. 10.1038/466304a 20631769

[36] Renner R, Thacker PD, Lubick N, Patel-Predd P, Christen K. Exxon Valdez oil no longer a threat? Environ Sci Technol. 2006; 40(20): 6188–6194. 17120536

[37] Li H, Boufadel MC. Long-term persistence of oil from the Exxon Valdez spill in two-layer beaches. Nat Geosci. 2010; 3(2): 96–99.

[38] Culbertson JB, Valiela I, Pickart M, Peacock EE, Reddy CM. Long‐term consequences of residual petroleum on salt marsh grass. J Appl Ecol. 2008; 45(4): 1284–1292.

[39] Chanton J, Zhao T, Rosenheim BE, Joye SB, Bosman S, Brunner CA, et al Using natural abundance radiocarbon to trace the flux of tetrocarbon to the seafloor following the Deepwater Horizon oil spill. Environ Sci Technol. 2014; 49(2): 847–854. 10.1021/es5046524 25494527

[40] Schwing PT, Romero IC, Brooks GR, Hastings DW, Larson RA, Hollander DJ. A decline in benthic foraminifera following the Deepwater Horizon event in the northeastern Gulf of Mexico. PloS One. 2015; 10(3): e0120565 10.1371/journal.pone.0120565 25785988 PMC4364910

[41] Boufadel MC, Sharifi Y, Van Aken B, Wrenn BA, Lee K. Nutrient and oxygen concentrations within the sediments of an Alaskan beach polluted with the Exxon Valdez oil spill. Environ Sci Technol. 2010; 44(19): 7418–7424. 10.1021/es102046n 20809617

[42] Culbertson JB, Valiela I, Peacock EE, Reddy CM, Carter A, VanderKruik R. Long-term biological effects of petroleum residues on fiddler crabs in salt marshes. Mar Pollut Bull. 2007; 54(7): 955–962. 17448504 10.1016/j.marpolbul.2007.02.015

[43] Khanna S, Santos MJ, Ustin SL, Koltunov A, Kokaly RF, Roberts DA. Detection of salt marsh vegetation stress and recovery after the Deepwater Horizon oil spill in Barataria Bay, Gulf of Mexico using AVIRIS data. PloS One. 2013; 8(11): e78989 10.1371/journal.pone.0078989 24223872 PMC3818498

[44] Dunton KH. Production ecology of *Ruppia maritima* L. sl and *Halodule wrightii* Aschers, in two subtropical estuaries. J Exp Mar Bio Ecol. 1990; 143(3): 147–164.

[45] McCall DD, Rakocinski CF. Grass shrimp (*Palaemonetes* spp.) play a pivotal trophic role in enhancing *Ruppia maritima* . Ecology. 2007; 88(3): 618–624. 17503590 10.1890/06-0375

[46] Cho HJ, Biber P, Nica C. The rise of *Ruppia* in seagrass beds: changes in coastal environment and research needs Handbook on environmental quality. Nova Science, New York, NY; 2009 pp.1–15.

[47] La Peyre MK, Rowe S. Effects of salinity changes on growth of *Ruppia maritima* L. Aquat Bot. 2003; 77(3): 235–241.

[48] Kanouse S, La Peyre MK, Nyman J. Nekton use of *Ruppia maritima* and non-vegetated bottom habitat types within brackish marsh ponds. Mar Ecol Prog Ser. 2006; 327: 61–69.

[49] Orth RJ, Moore KA. Distribution of *Zostera marina* L. and *Ruppia maritima* L. sensu lato along depth gradients in the lower Chesapeake Bay, USA. Aquat Bot. 1988; 32(3): 291–305.

[50] Valinoti CE, Ho CK, Armitage AR. Native and exotic submerged aquatic vegetation provide different nutritional and refuge values for macroinvertebrates. J Exp Mar Bio Ecol. 2011; 409(1): 42–47.

[51] Agami M, Waisel Y. The role of fish in distribution and germination of seeds of the submerged macrophytes *Najas marina* L. and *Ruppia maritima* L. Oecologia. 1988; 76(1): 83–88. 28312383 10.1007/BF00379604

[52] Mendelssohn IA, Hester MW, Sasser C, Fischel M. The effect of a Louisiana crude oil discharge from a pipeline break on the vegetation of a southeast Louisiana brackish marsh. Oil and Chem Pollut. 1990; 7(1): 1–15.

[53] Rodríguez‐Pérez H, Green AJ. Waterbird impacts on widgeongrass *Ruppia maritima* in a Mediterranean wetland: comparing bird groups and seasonal effects. Oikos. 2006; 112(3): 525–534.

[54] Horel A, Mortazavi B, Sobecky PA. Responses of microbial community from northern Gulf of Mexico sandy sediments following exposure to deepwater horizon curde oil. Environ Toxicol Chem. 2012; 31(5): 1004–1011. 10.1002/etc.1770 22447770

[55] Ortmann AC, Anders J, Shelton N, Gong L, Moss AG, Condon RH. Dispersed oil disrupts microbial pathways in pelagic food webs. PloS One. 2012; 7(7): e42548 10.1371/journal.pone.0042548 22860136 PMC3409195

[56] Turner RE, Overton EB, Meyer BM, Miles MS, McClenachan G, Hooper-Bui L, et al Distribution and recovery trajectory of Macondo (Mississippi Canyon 252) oil in Louisiana coastal wetlands. Mar Pollut Bull. 2014a; 87(1): 57–67. 25176275 10.1016/j.marpolbul.2014.08.011

[57] Turner RE, Overton EB, Meyer BM, Miles MS, Hooper-Bui L. Changes in the concentration and relative abundance of alkanes and PAHs from the Deepwater Horizon oiling of coastal marshes. Mar Pollut Bull. 2014b; 86(1): 291–297. 25127500 10.1016/j.marpolbul.2014.07.003

[58] Martin CW, Valentine JF. Eurasian milfoil invasion in estuaries: physical disturbance can reduce the proliferation of an aquatic nuisance species. Mar Ecol Prog Ser. 2012; 449: 109–119.

[59] Martin CW, Valentine JF. Sexual and asexual reproductive strategies of invasive Eurasian milfoil (*Myriophyllum spicatum*) in estuarine environments. Hydrobiol. 2014; 727(1): 177–184.

[60] Puntila RI, Martin CW, Valentine JF. Measuring predation with a new design of submersible chronographic timer. Bull Mar Sci. 2012; 88(4): 1115–1122.

[61] Sokal RR, Rolf FJ. Biometry: the principals and practice of statistics in biology research. WF Freeman New York; 1995.

[62] Silliman BR, van de Koppel J, McCoy MW, Diller J, Kasozi GN, Earl K, et al Degradation and resilience in Louisiana salt marshes after the BP—Deepwater Horizon oil spill. Proc Natl Acad Sci U S A. 2012; 109(28): 11234–11239. 10.1073/pnas.1204922109 22733752 PMC3396483

[63] McCall BD, Pennings SC. Disturbance and recovery of salt marsh arthropod communities following BP Deepwater Horizon oil spill. PloS One. 2012; 7(3): e32735 10.1371/journal.pone.0032735 22412916 PMC3296729

[64] Fodrie FJ, Able KW, Galvez F, Heck KL, Jensen OP, López-Duarte PC, et al Integrating organismal and population responses of estuarine fishes in Macondo spill research. BioScience. 2014; 64(9): 778–788.

[65] Able KW, López-Duarte PC, Fodrie FJ, Jensen OP, Martin CW, Roberts BJ, et al Fish assemblages in Louisiana salt marshes: effects of the Macondo oil spill. Estuaries Coast. 2015; 38(5): 1385–1398. 10.1007/S12237-014-9890-6

[66] McClenachan G, Turner RE, Tweel AW. Effects of oil on the rate and trajectory of Louisiana marsh shoreline erosion. Environ Res Lett. 2013; 8(4): 044030.

[67] Phillips RC, Grant WS, McRoy CP. Reproductive strategies of eelgrass (*Zostera marina* L.). Aquat Bot. 1983; 16(1): 1–20.

[68] Harrison PG, Durance C. Variation in clonal structure in an eelgrass (*Zostera marina*) meadow on the Pacific coast of Canada. Can J Bot. 1992; 70(3): 653–657.

[69] Conacher CA, Poiner IR, Butler J, Pun S, Tree DJ. Germination, storage and viability testing of seeds of *Zostera capricorni* Aschers from a tropical bay in Australia. Aquat Bot. 1994; 49(1): 47–58.

[70] Graham WM, Condon RH, Carmichael RH, D’Ambra I, Patterson HK, Linn LJ, et al Oil carbon entered the coastal planktonic food web during the Deepwater Horizon oil spill. Environ Res Lett. 2010; 5(4): 045301.

[71] Quintana-Rizzo E, Torres JJ, Ross SW, Romero I, Watson K, Goddard E, et al δ 13 C and δ 15 N in deep-living fishes and shrimps after the Deepwater Horizon oil spill, Gulf of Mexico. Mar Pollut Bull. 2015; 94(1–2): 241–250. 10.1016/j.marpolbul.2015.02.002 25778549

[72] Hastings DW, Schwing PT, Brooks GR, Larson RA, Morford JL, Roeder T, et al Changes in sediment redox conditions following the BP DWH blowout event. Deep Sea Res Part II: Top Stud Oceanogr. 2015;

[73] Pedersen O, Borum J, Duarte C, Mand Fortes MD. Oxygen dynamics in the rhizosphere of *Cycomocea rotundata* . Mar Ecol Prog Ser. 1998; 169: 283–288.

[74] Connell EL, Colmer TD, Walker DI. Radial oxygen loss from intact roots of *Halophila* ovalis as a function of distance behind the root tip and shoot illumination. Aquat Bot. 1999; 63(3): 219–228.

[75] Greve TM, Borum J, Pedersen O. Meristematic oxygen variability in eelgrass (*Zostera marina*). Limnol Oceanogr. 2003; 48(1): 210–216.

[76] McNutt MK, Camilli R, Crone TJ, Guthrie GD, Hsieh PA, Ryerson TB, et al Review of flow rate estimates of the Deepwater Horizon oil spill. Proc Natl Acad Sci U S A. 2012; 109(50): 20260–20267. 10.1073/pnas.1112139108 22187459 PMC3528583

[77] Valentine DL, Fisher GB, Bagby SC, Nelson RK, Reddy CM, Sylva SP, et al Fallout plume of submerged oil from Deepwater Horizon. Proc Natl Acad Sci U S A. 2014; 111(45): 15906–15911. 10.1073/pnas.1414873111 25349409 PMC4234598

