# Supplementary figures and images for: Effects of Oil-Contaminated Sediments on Submerged Vegetation: An Experimental Assessment of Ruppia maritima
Source: PLoS One. 2015 Oct 2;10(10):e0138797. doi: 10.1371/journal.pone.0138797 (PMC4592016; doi:10.1371/journal.pone.0138797)

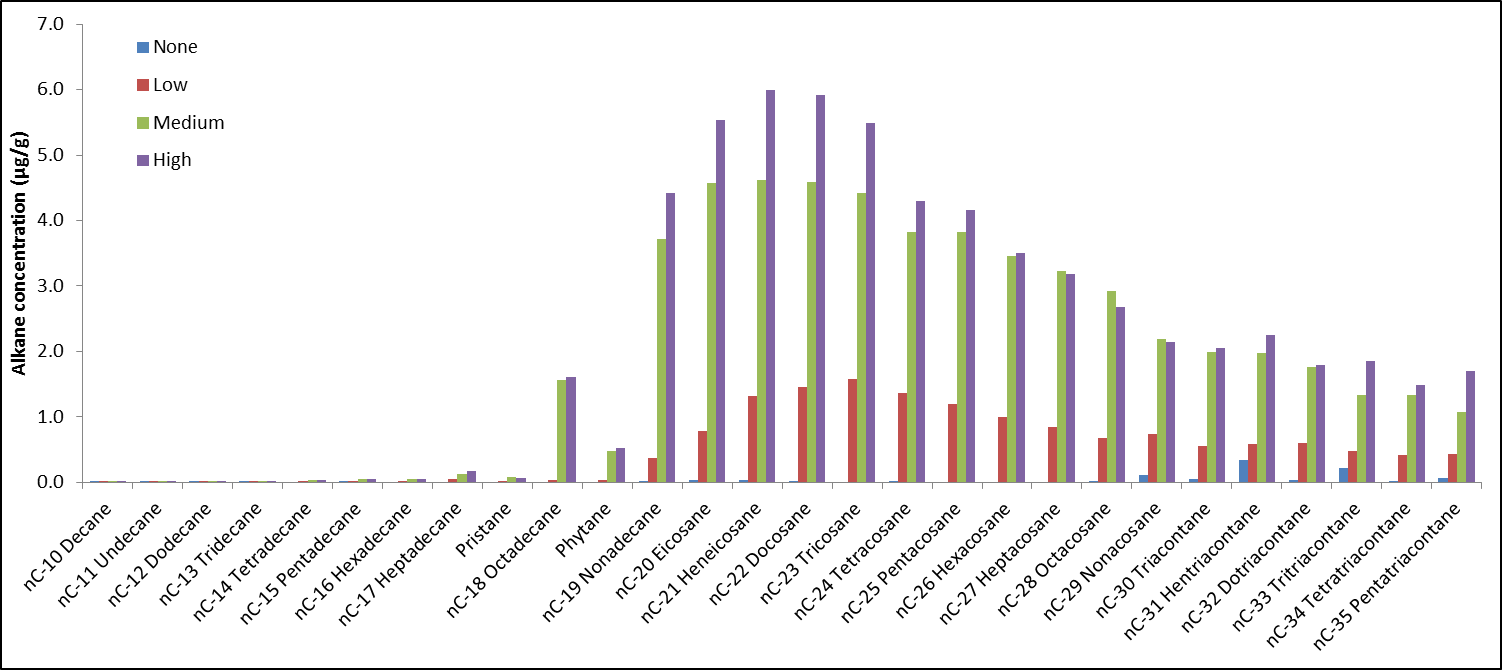

Supplement: S1 Fig — The concentration of alkane petroleum hydrocarbons (μg g-1) measured at the end of the experiment. (TIF) [file pone.0138797.s001.tif]

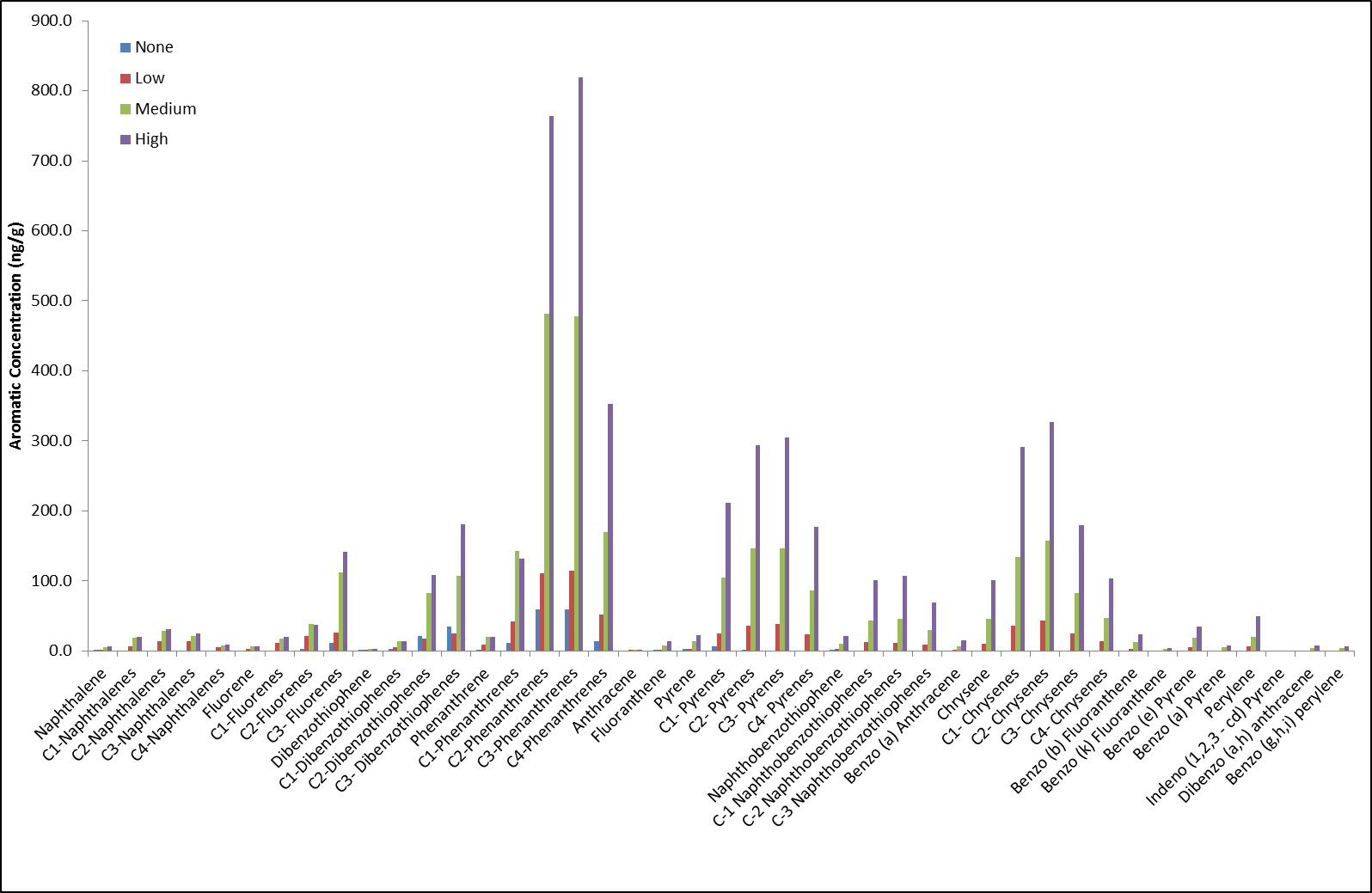

Supplement: S2 Fig — The concentration of aromatics petroleum hydrocarbons (ng g-1) measured at the end of the experiment. (TIF) [file pone.0138797.s002.tif]
